# Improved elongation factor-1 alpha-based vectors for stable high-level expression of heterologous proteins in Chinese hamster ovary cells

**DOI:** 10.1186/1472-6750-14-56

**Published:** 2014-06-14

**Authors:** Nadezhda A Orlova, Sergey V Kovnir, Julia A Hodak, Ivan I Vorobiev, Alexandre G Gabibov, Konstantin G Skryabin

**Affiliations:** 1 Laboratory of Mammalian Cell Bioengineering, Centre “Bioengineering”, Russian Academy of Sciences, 60-letija Oktyabrya 7, Moscow 117312, Russia; 2 Laboratory of Biocatalysis, Institute of Bioorganic Chemistry, Russian Academy of Sciences, Miklukho-Maklaya 16/10, Moscow 119971, Russia; 3 Kazan Federal University, Kazan, Republic of Tatarstan 420008, Russia

**Keywords:** CHO cells, High level expression, Stable cell line generation, Molecular cloning

## Abstract

**Background:**

Establishing highly productive clonal cell lines with constant productivity over 2–3 months of continuous culture remains a tedious task requiring the screening of tens of thousands of clonal colonies. In addition, long-term cultivation of many candidate lines derived in the absence of drug selection pressure is necessary. Expression vectors based on the elongation factor-1 alpha (EEF1A) gene and the dihydrofolate reductase (DHFR) selection marker (with separate promoters) can be used to obtain highly productive populations of stably transfected cells in the selection medium, but they have not been tested for their ability to support target gene amplification under gradually increasing methotrexate pressure.

**Results:**

We have modified EEF1A-based vectors by linking the DHFR selection marker to the target gene in the bicistronic RNA, shortening the overall plasmid size, and adding an Epstein-Barr virus terminal repeat fragment (EBVTR) element. Presence of the EBVTR element increased the rate of stable transfection by the plasmid by 24 times that of the EBVTR-minus control and improved the rate of methotrexate-driven gene amplification. The mean expression level of the enhanced green fluorescent protein (eGFP) used herein as a model protein, increased up to eight-fold using a single round of amplification in the case of adherent colonies formation and up to 4.5-fold in the case of suspension polyclonal cultures. Several eGFP-expressing cell populations produced using vectors with antibiotic resistance markers instead of the DHFR marker were compared with each other. Stable transfection of Chinese hamster ovary (CHO) DG44 cells by the p1.2-Hygro-eGFP plasmid (containing a hygromycin resistance marker) generated highest eGFP expression levels of up to 8.9% of the total cytoplasmic protein, with less than 5% of the cell population being eGFP-negative.

**Conclusions:**

The p1.1 vector was very effective for stable transfection of CHO cells and capable of rapid MTX-driven target gene amplification, while p1.2-Hygro achieved similar eGFP expression levels as p1.1. The set of vectors we have developed should speed-up the process of generating highly productive clonal cell lines while substantially decreasing the associated experimental effort.

## Background

Most of the proteins currently employed for therapeutic use are produced by stably transfected mammalian cells, of which the most popular is the Chinese hamster ovary (CHO) cell line. Establishing highly productive clonal cell lines that exhibit constant productivity over a 2–3 month period of continuous culture remains a tedious task, requiring tens of thousands of clonal colonies to be screened, followed by the long-term cultivation of candidate lines in the absence of an appropriate selection pressure. Generally, the expression levels of a target gene may be increased by its amplification in the genome [1], which is usually achieved by linking the target gene to the murine dihydrofolate reductase (DHFR) gene with stepwise increases in the concentration of the DHFR inhibitor, methotrexate (MTX), in the selection medium.

Target gene amplification is a time-consuming process, resulting in cell populations that often contain unstable clones, and in the absence of an appropriate selection pressure, reduced production levels. The probability of obtaining a highly productive clonal cell line can be increased significantly by utilizing plasmids based on non-coding parts of the elongation factor-1 alpha gene (EEF1A) from Chinese hamster, as described by Running Deer and Allison [2]. Expression vector pDEF38, introduced by these authors, differs significantly from the widely employed vectors with the core promoter of the human ortholog elongation factor 1 alpha gene (EF1a). EEF1A-based expression vector includes 4.1 kb upstream and 4.2 kb downstream flanking areas of the EEF1A gene, so the ORF of the of the target gene replaces the coding exons of the elongation factor 1 alpha protein in the natural EEF1A gene, mimicking with all possible accuracy the structure of the natural gene in the resulting expression plasmid. It was shown that presence of both flanking areas in the EEF1A-based vectors results in the 6- to 35- fold increase of the average expression level comparing to commercial vectors with CMV or EF1alpha promoters. Removal of the downstream flanking area from the expression vector resulted in the 4-fold drop in the expression level.

Original expression vector pDEF38 contained the DHFR selection marker with a separate SV40 promoter and was not tested for its ability to support target gene amplification under gradually increasing MTX pressure. DHFR-compatible vectors, bearing the neomycin resistance gene instead of the DHFR gene, were also described in the same work. Existing EEF1A-based vectors, despite their high promoter strength and their long-term production level stability, do not accommodate very large plasmid sizes. Consequently, this can result in low-level genome integration and inability to maintain the target gene amplification step, possibly as a result of vector fragmentation and autonomous amplification of the DHFR-coding area.

Since EEF1A-based vectors are much longer than CMV-based vectors, they are expected to have lower transfection efficiency and, subsequently, lower numbers of stably transfected cells. It was shown, that the insertion the concatemer fragment of the Epstein-Barr virus terminal repeats (EBVTR) [3,4] in the expression vectors increase the rate of stably transfected colonies formation by five to ten fold [5]. The molecular mechanism of this effect is poorly understood. It is known that G-rich repeats in the EBVTR bind to the cellular protein terminal repeat binding protein (TRBP) [3] and at least two binding sites of TRBP were identified in the repetitive cellular DNA [6]. EBVTR areas are involved in the integration of the Epstein-Barr virus into the chromosomal DNA [7]. EBV-infected cells may harbour the virus in the chromosome-integrated form, as the independently replicating episome or the mixture of both forms [8]. Region of the EBV, called oriP, maintains the episomal replication of the EBV genome, interacts with the EBV-encoded nuclear antigen-1 (EBNA-1) and allows EBV plasmids to separate in mitosis through binding to chromosomes [9]. EBVTR concatemer used for enhancement of expression plasmids, however, contains no sequences from the oriP region and no DNA fragments with significant homology toward oriP region, so the EBNA-1 – mediated persistence of the EBVTR-containing plasmid as the episome in the transfected cells is highly unlikely.

We hypothesized that significant improvements to EEF1A-based vectors might be achieved by: 1) inserting the EBVTR element outside of the EEF1A flanking DNA; 2) linking the DHFR open reading frame to the target gene by the internal ribosome entry site (IRES) thereby preventing the possibility of separate amplification of the selection marker; 3) reducing of the length of the backbone DNA, which is necessary for maintaining the plasmid in the bacterial host. Similar improvements might be applied to DHFR-compatible EEF1A-based vectors used for monocistronic expression of a target gene; in this case by placing the antibiotic resistance genes outside the context of the non-coding parts of the elongation factor 1 alpha gene, which might decrease genetic linkage between the selection marker and the target gene.

Here, we report on the functional properties of EEF1A-based plasmid vectors for bicistronic and monocistronic expression. We also describe the corresponding methods for obtaining highly productive and stable cell lines that maintain constant productivity levels after genome amplification of the integrated plasmid, utilizing the DHFR-negative cell line CHO DG44 [10,11]. In addition, we used the enhanced green fluorescent protein (eGFP) as a model target protein, and show eGFP accumulation inside CHO cells.

## Methods

### Molecular cloning

The sequences of the primers used for cloning expression plasmids are shown in Additional file 1: Table S1. The backbone vector, pBL-2, was obtained by two stages of inverted PCR using long adapter primers and the pUC18 plasmid as a template. Non-functional parts of the plasmid including the pLac promoter and the LacZ gene were removed. Inverted PCR was performed as described previously [12]. Oligonucleotides and PCR reagents were from Evrogen, JSC (Moscow, Russia). PCR products were purified from 1% agarose gels by the Wizard SV Gel and PCR Clean-Up System (Promega, Madison, WI). T4 polynucleotide kinase and *Mal*I restriction endonuclease (Sibenzyme, Novosibirsk, Russia) were used. T4 DNA ligase (Fermentas, Vilnius, Lithuania) was used for inverted PCR product circularization. The *Escherichia coli* TOP10 strain (Invitrogen, Carlsbad, CA) was used for cloning. Plasmids were isolated with a GeneJet Plasmid Purification Kit (Fermentas, Vilnius, Lithuania).

The pAL-EBV plasmid, containing a fragment of a concatemer of EBV terminal repeats, as described previously [5] and nearly undistinguishable from the human herpes virus 4 strain K4123-MiEBV sequence [GenBank: KC440852.1] was made from synthetic oligonucleotides cloned into a pAL-TA (Evrogen) vector. The ORF encoding mouse DHFR was obtained by PCR using pOptivec-TOPO linearized vector (Invitrogen) as a template. The fragment encoding the attenuated encephalomyocarditis virus (EMCV) IRES was obtained from the pOptivec-circ plasmid (self-ligated pOptivec-TOPO) by restriction. These fragments were cloned into the pBL-2 plasmid via assembly of two different intermediate constructs, PBL-2-ID and PBL-2-ID-EBV. DNA modification enzymes for routine molecular cloning were obtained from Fermentas or Sibenzyme.

### Construction of p1.1 vectors

**Figure 1:**
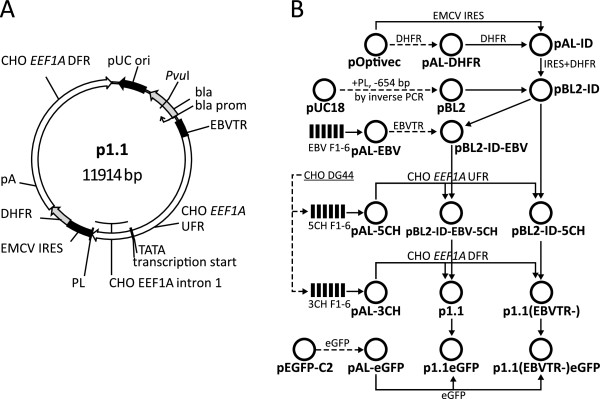
**Map of the p1.1 plasmid vector and the cloning scheme for p1.1-based plasmids. A**. Plasmid map. UFR: upstream flanking region of the EEF1A gene; DFR: downstream flanking region; PL: polylinker region; pA: polyadenylation signal; bla – ampicillin resistance gene; bla prom – promoter of the ampicillin resistance gene. **B**. Cloning scheme for p1.1-based plasmids. Generation of cloning inserts by PCR with adaptor primers is depicted by dashed lines; generation of cloning inserts by restriction is depicted by solid lines. EBV F1-6: corresponding synthetic fragments of the EBVTR element. 5CH F1-6: corresponding fragments of the upstream flanking region of the EEF1A gene; 3CH F1-6: corresponding fragments of the downstream flanking region of the EEF1A gene.

Fragments corresponding to the upstream and downstream flanking regions (8532–12603 and 14545–18794 sequences of [GenBank:AY188393]) of the CHO elongation factor 1 gene were obtained by PCR using CHO DG44 cell (Invitrogen) genomic DNA as a template. The modular assembly cloning technique used herein is described in detail elsewhere [13].Assembled CHO genomic regions were cloned into the intermediate plasmids, PBL-2-ID and PBL-2-ID-EBV, resulting in p1.1(EBVTR-) and p1.1 expression vectors, respectively (Figure 1).

### Construction of p1.2 vectors

**Figure 2:**
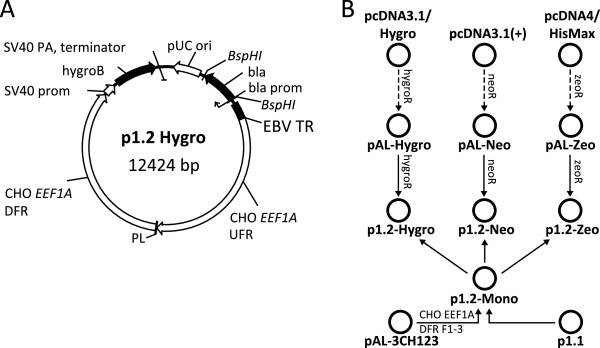
**Map of the p1.2-Hygro plasmid vector and the cloning scheme for p1.2-based plasmids. A**. Plasmid map. UFR: upstream flanking region of the EEF1A gene; DFR: downstream flanking region; Pl: polylinker region; SV40 prom and SV40 PA: promoter and polyadenylation signal from the SV40 virus. **B**. Cloning scheme for p1.2-based plasmids.

p1.2-Mono, the intermediate backbone plasmid for expression vectors bearing antibiotic resistance genes was obtained by removal of the region containing the EMCV IRES and the DHFR ORF from the p1.1 expression vector. Plasmid pAL-3CH123, containing first three modules of the downstream flanking region of the *EEF1A* was used as the source of the donor DNA insert fragment, replacing the deleted IRES and DHFR area, so both flanking regions of the *EEF1A* remained unaltered (Figure 2). Antibiotic resistance genes and the SV40 promoter and terminator regions were obtained by amplification with adaptor primers, using pcDNA3.1/Hygro, pcDNA3.1(+), and self-ligated pcDNA4/HisMax-TOPO (Invitrogen) as PCR templates. Antibiotic resistance cassettes were sub-cloned into T-vectors and then transferred into the p1.2-Mono backbone by restriction-ligation resulting in p1.2-Hygro, p1.2-Neo and p1.2-Zeo. A DNA fragment encoding eGFP and a consensus Kozak sequence (GCCGCCATGG) [14] was obtained by PCR with adaptor primers and the pEGFP-C2 plasmid (Clontech, Mountain View, CA) as a template and then cloned into the polylinker area of p1.1 and p1.2 vectors, thereby resulting in p1.1(EBVTR-)eGFP, p1.1eGFP, p1.2-HygroeGFP, p1.2-NeoeGFP and p1.2-ZeoeGFP expression plasmids.

Purified plasmids for transfection and the control plasmid pEGFP-N2 (Clontech) were prepared using an EndoFree Plasmid Maxi Kit (Qiagen, Valencia, CA). For stable cell line generation all plasmids except p1.2-HygroeGFP were linearized by restriction with *Pvu*I by cutting inside the ampicillin resistance gene *bla* sequence. The plasmid p1.2-HygroeGFP was restricted with *BspH*I, which introduced two breaks near the *bla* gene.

### Cell culture

A DHFR-negative CHO DG44 cell line (Invitrogen) was cultured in shaking flasks in the chemically defined medium, CD DG44 (Invitrogen), supplemented with 0.18% Pluronic F-68 (Invitrogen) and 4 mM L-glutamine (Invitrogen). The cells were passaged 24 h before transfection. For direct colony generation in 96-well culture plates, transfection was performed using Fugene HD reagent (Promega), containing 60 μg of DNA and 180 μl of the reagent per 15 millions of cells in 30 ml of the above medium. Plasmids p1.2 were transfected by electroporation in Gene Pulser Electroporation Buffer (Bio-Rad, Hercules, CA) using a cuvette with a 4 mm gap with 7.5 million cells and 15 μg of linearized DNA for each transfection. Cells were counted by trypan blue exclusion and fluorescence microscopy at 48 h post-transfection. For direct generation of colonies, transiently transfected cell cultures were transferred into CHO-A culture medium (Invitrogen) lacking hypoxanthine and thymidine (HT), and seeded at 5000 cells/well in the culture plates. Cells were grown undisturbed for 14 days and inspected by fluorescence microscopy. The medium was changed and the plates were cultivated for 5–10 additional days until the first 10% of the wells containing colonies became confluent.

To generate stably transfected cell populations using p1.1eGFP and p1.1(EBVTR-)eGFP plasmids, transiently transfected cultures were transferred to OptiCHO medium (Invitrogen) lacking HT, and thereafter cultivated in shaking flasks with medium exchange every 3 days until the cell viability increased to 85% (approximately 22–27 days of cultivation).

MTX-driven target gene amplification in culture plates was performed by seeding the cells from stably transfected cell populations into 96-well culture plates at a density of 5000 cells/well in the CHO-A culture medium, supplemented with 0, 50, 100, 200, 400 or 800 nM MTX. Three plates were used for each concentration of MTX. The cells were grown undisturbed for 14 days, after which the plates were inspected by microscopy and the culture medium was changed every 4 days until the first 10% of wells in each plate became confluent. Plates were screened again by fluorescence microscopy, and cells from the 16 brightest wells from each plate were transferred into a 48-well plate, grown to confluence, and then transferred into 24-well plates. Colonies lacking normal proliferation speeds or attached to the surface of the plates too tightly for dislodging by pipetting were discarded. Cells from the eight brightest wells for each MTX concentration were dislodged from their plates, lysed as described below, and then used to determine eGFP levels. Six randomly picked colonies, obtained in the presence of 400 and 800 nM MTX, were transferred into a 6-well plate and grown with shaking in OptiCHO medium with passages made every three days for 60 days. Samples for eGFP level determination were collected every second passage.

**Figure 3:**
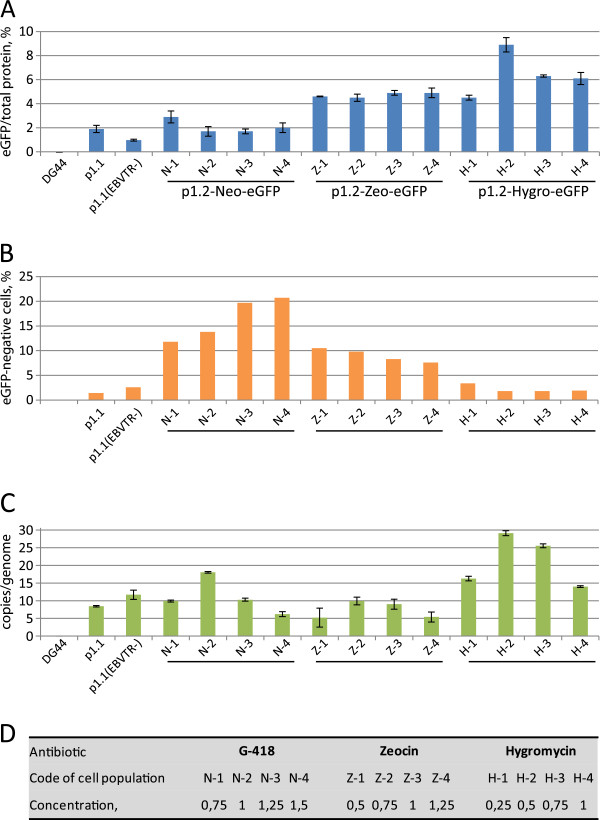
**Properties of the cell populations stably transfected by p1.2-based plasmids under various drug selection stringencies.** DG44: untransfected CHO DG44 cells; p1.1: cells stably transfected by the p1.1eGFP plasmid and selected in the presence of 200 nM MTX; p1.1(EBVTR-): transfection by the p1.1(EBVTR-)eGFP plasmid using the same conditions. **A**. Level of intracellular eGFP in cell populations. Error bars indicate the standard deviation, n = 2. **B**. Proportion of eGFP-negative cell populations measured by FACS. **C**. Number of copies of genome-integrated plasmids measured by Q-PCR. Amplicons are located inside the eGFP ORF and one representative value experiment from three independent measurements is shown. Error bars represents standard deviations, n = 3-4. The apparent level of the eGFP ORF DNA for the untransfected CHO DG44 cells is below 0.1 copies per one haploid genome. **D**. Codes for the different cell populations and the concentrations of antibiotics employed.

Target gene amplification for polyclonal cell populations was performed for the suspension culture of CHO DG44 cells, stably transfected by the p1.1eGFP plasmid in presence of 50 nM MTX, as described above. Concentration of the MTX in the culture medium was increased by two-fold steps, each after two consecutive passages, until the cell viability decreased below 85%. Resulting culture, obtained in presence of 0.8 μM MTX, was split into four flasks, supplemented by 0.8; 1.6; 3.2; 6.4 μM MTX and cultured until the cell viability returned to at least 85% (7–12 days).Generation of polyclonal cell populations involving transfected p1.2 plasmids were performed by seeding transiently transfected cells in 6-well culture plates, using 1 million of viable cells per well in 5 ml of DG44 medium, supplemented with the corresponding antibiotic, or 5 ml of OptiCHO medium with 200 nM MTX for control transfections using p1.1 plasmids. The concentrations of the antibiotics used are shown in Figure 3. Plates were cultivated with shaking until the cell viability returned to at least 85% (20 days), after which the medium was changed every 4 days.

### Determination of eGFP concentrations in cell lysates

Cell culture samples containing approximately one million of cells were centrifuged and the cell pellets were resuspended in phosphate buffered saline and recentrifuged. The washed cell pellets were resuspended in 0.1 ml of lysis solution containing 150 mM NaCl, 50 mM Tris–HCl at pH 7.5, 1% Triton X-100, a protease inhibitor cocktail (Sigma, St. Louis, MO), and then incubated for 30 min on ice with stirring. Cell debris was removed by centrifugation. The concentration of eGFP in the lysate from the H-4 cell population (Figure 3) was measured by spectrophotometry at a wavelength of 488 nm using a molar extinction coefficient of 55,000 M^−1^ · cm^−1^ and an eGFP molecular weight of 32.7 kDa [15]. The fluorescence intensity of eGFP in all of the lysates was measured along with the serially diluted calibration samples, which were prepared from the H-4 lysate containing a known concentration of eGFP. Total protein concentration in the lysates was measured by the Bradford method with bovine serum albumin as a standard.

### FACS analysis and quantitative PCR

Undiluted cell culture samples were subject to FACS FC 500 (Beckman Coulter, Krefeld, Germany) analysis at an emission at 488 nm and detection through a 530/40-nm bandpass filter. At least 10,000 individual cells were counted for each sample analysed. Quantitative PCR analysis of the expression plasmid copy numbers in the genomes of stably transfected cells was performed using an iCycler iQ thermocycler (Bio-Rad) and qPCRmix-HS SYBR (Evrogen) reaction mixture with the primers shown in Additional file 1: Table S2. The highly purified p1.1eGFP plasmid was used as a quantity calibrator using five different concentrations for each determination performed in triplicate. PCR was performed three times with three to four replicates for each sample. Genomic DNA was extracted from cells with a Genomic DNA Purification Kit (Fermentas) and quantified using a Qubit fluorometer (Invitrogen) and the dsDNA HS kit (Invitrogen). Quantity calibrator plasmid was used as the external standard for the quantification of genomic DNA samples by fluorometry.

## Results and discussion

### Construction of expression plasmids

Since the transfection efficiency and, probably, the genome integration rate of an expression plasmid is inversely proportional to its size [16], we made a minimal backbone plasmid by eliminating most of the unnecessary elements from the pUC18 plasmid. The resulting plasmid, pBL-2, lacks the f1 origin of replication, and the bacterial promoter of the LacZ gene along with the LacZ ORF itself and some flanking DNA regions. Overall, the resulting plasmid length decreased some 600 bp from 2686 to 2032 bp.

The upstream and downstream regions of the EEF1A gene were obtained from CHO DG44 cell genomic DNA using the modular assembly cloning technique described previously [13]. A concatemer of terminal repeats from the Epstein-Barr virus (EBVTR) [3,4] was assembled from synthetic oligonucleotides using the same technique and was inserted along with the IRES from the encephalomyocarditis virus and the murine DHFR open reading frame into the pBL-2 vector. Cloning the upstream and downstream flanking areas of the EEF1A gene into the pBL-2-ID-EBV plasmid resulted in the expression vector p1.1 (Figure 1). A control vector, lacking the EBVTR fragment, was assembled similarly and is denoted here as p1.1(EBVTR-). The p1.1 plasmid was approximately 1.5 kbp shorter than the original EEF1A-based plasmid, pDEF38, despite addition of the EBVTR fragment. The eGFP ORF with the synthetic consensus Kozak sequence [14] was cloned into both vectors and the resulting plasmids p1.1eGFP and p1.1(EBVTR-)eGFP were used for CHO cell transfections.

### Generation of stably transfected colonies using p1.1-based plasmids

Transient transfection of the DHFR-deficient CHO DG44 cells resulted in significantly decreased transfection efficiencies for both of the EEF1A-based plasmids relative to the cytomegalovirus (CMV)- promoter-based 4700 bp pEGFP-N2 plasmid, and approximately the same transfection efficiencies and eGFP expression levels for plasmids with or without the EBVTR element (Table 1).

**Table 1:** Properties of the transiently transfected cells used in this study

**Plasmid name**	**pEGFP-N2**	**p1.1eGFP**	**p1.1(EBVTR-)eGFP**
eGFP-expressing cells	22.8%	5.8%	6.0%
Fluorescence intensity, RFU/50 cells	114	35.3	32.0
Viable cells	86%	83%	84%

**Figure 4:**
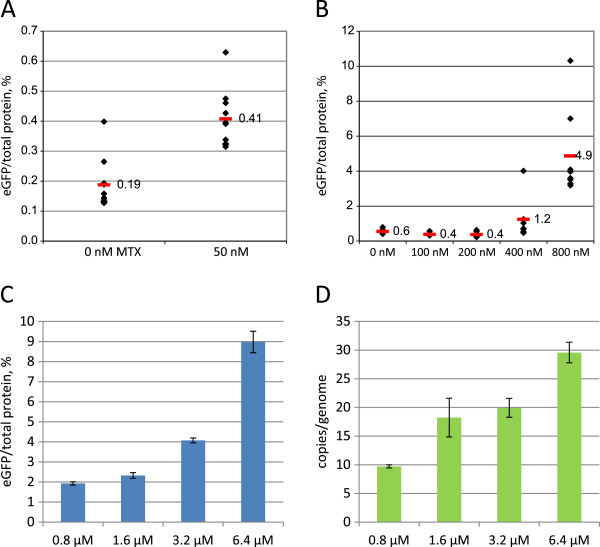
**eGFP-expressing cell colonies obtained by elevated selection pressure and by targeted gene amplification.** Red bars: mean eGFP levels for sets of cell colonies analysed. Concentrations represent final concentrations of MTX used. **A**. eGFP levels for 10 colonies obtained in the absence of MTX and in the presence of 50 nM MTX, colonies were obtained by the direct plating of transiently transfected cells. **B**. eGFP levels for 8 colonies obtained by growth in the presence of various MTX concentrations. Polyclonal stably transfected cell population was used for plating. **C**. Level of intracellular eGFP in polyclonal cell populations, obtained by primary selection in presence of 50 nM MTX and subsequent amplification in presence of various concentrations of MTX. Error bars indicate the standard deviation, n = 2. **D**. Number of copies of genome-integrated plasmids measured by Q-PCR for populations from panel C. Amplicons are located inside the eGFP ORF and one representative value experiment from three independent measurements is shown. Error bars represents standard deviations, n = 3.

At the same time, stable integration rate (or rate of establishment of stable episomal maintenance) of the p1.1eGFP plasmid was 24 times higher than that of the p1.1(EBVTR-)eGFP control plasmid in the selection medium lacking both HT and MTX (Table 2), clearly indicating that the EBVTR element was active in the very large expression plasmid. Transfection and selection of stably transformed CHO DG44 cells by the p1.1eGFP plasmid was repeated with the selection medium supplemented with 50 nM MTX. In this case, the eGFP expression level increased twice for the ten most productive wells (Figure 4A). Thus, the p1.1 plasmid is suitable for creation of stably transfected cell clones or populations under variable selection stringencies.

**Table 2:** Colony formation efficiency for p1.1eGFP and p1.1(EBVTR-)eGFP plasmids

**Plasmid name**	**p1.1eGFP**	**p1.1(EBVTR-)eGFP**
Total number of colonies in 10 culture plates	2342	95
eGFP-expressing colonies in 10 culture plates and their proportions	2093 (89.4%)	52 (54.7%)
Fluorescence intensity of the brightest well, RFU/50 cells	210	45.1

### MTX-driven target gene amplification

Since the EBVTR element was effective at increasing the incidence of stable transfection, we tested its ability to speed up the transgene amplification process. Polyclonal populations of CHO DG44 cells, transfected by p1.1eGFP and p1.1(EBVTR-)eGFP plasmids and selected for stable integration by suspension cultivation in the absence of MTX and HT, were seeded in the 96-well culture plates in the presence of 0–800 nM MTX and grown undisturbed until visible colonies developed. For the p1.1(EBVTR-)eGFP plasmid lacking the EBVTR element, no viable cell colonies were obtained in the presence of 200, 400 and 800 nM MTX. The eGFP expression levels of the highest expressing colonies, obtained in the presence of 0 and 100 nM MTX, was in the same range as the colonies obtained by direct plating of transiently transfected cells in the absence of MTX (data not shown). In the case of the p1.1-eGFP plasmid, multiple colonies were obtained for all of the concentrations of MTX tested. After visual screening by fluorescence microscopy and expansion, the eight brightest colonies for each concentration of MTX employed were grown to confluency in 24-well culture plates. The relative eGFP expression levels for these colonies (Figure 4B) was approximately 8 times higher when cultivated with 800 nM MTX, and approximately 2 times higher when cultivated with 400 nM MTX, compared with cultivation without MTX. Six randomly chosen colonies, obtained in the presence of 400 and 800 nM MTX, were scaled up, re-adapted to suspension culture and cultivated for 60 days. No substantial decay in the eGFP expression level was detected for each colony (data not shown). Thus, the p1.1 vector is suitable for target gene amplification in the presence of MTX. The resulting cell clones are sufficiently stable for cell bank generation and subsequent large-scale cultivation.Target gene amplification process was also tested for polyclonal cell population, obtained by the primary selection in the presence of 50 nM MTX. Sequential addition of MTX from 100 nM to 400 nM gave no decrease in cell viability, eGFP level was also constant (data not shown). Further addition of 0.8 – 6.4 μM of MTX, performed in one step for multiple culture flasks, resulted in the concentration-dependent increase of eGFP content (Figure 4C), peaking at 9% of the total protein in the case of 6.4 μM of MTX. Analysis of the copy numbers of the integrated plasmids using quantitative PCR (Figure 4D) showed that higher MTX level and higher eGFP content correspond to higher copy number of the integrated plasmid. Hereby, the vector p1.1 is suitable for obtaining highly productive cell populations or clones by direct clone selection in culture plates in the presence of MTX or by the multi-step target gene amplification in the suspension culture.

### Polyclonal cell populations stably transfected by p1.2 plasmids

**Figure 5:**
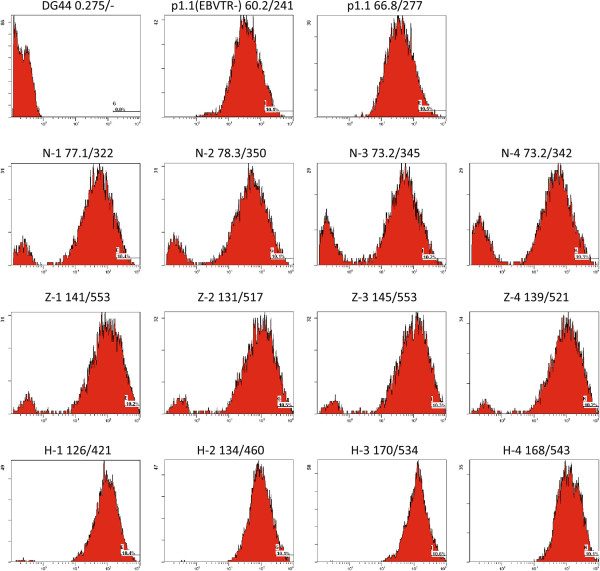
**Distribution of the eGFP expression levels in cell populations as determined by FACS analysis.** Codes for the corresponding cell populations are the same as in Figure 3. First number after the cell population code: mean level of eGFP in the sample; second number: mean level of eGFP in the upper 10% of the eGFP-positive cells.

Heterologous expression of a functionally active target protein often requires co-expression of a small number of protein processing enzymes. For example, the blood clotting factor IX expression systems used with CHO or BHK cells rely on co-expression of the signal protease PACE/furin [17] and the vitamin-K recharging enzyme, VKORC1 [18]. Generally, the expression levels of such “helper” proteins should be lower than that of the target protein, but of comparable magnitude. If the target protein is coded by a plasmid bearing a DHFR selection marker, helper proteins may be coded by plasmids with the same structure, but bearing antibiotic resistance markers. We have tested resistance markers for three widely used antibiotics, G418 (a neomycin analogue), zeocin, and hygromycin, in the EEF1A-based expression vector, which was modified by removing the IRES fragment and the DHFR open reading frame from the p1.1 plasmid, and insertion of the corresponding antibiotic resistance genes outside of the EEF1A gene flanking areas and controlled by a separate SV40 promoter. The resulting vectors, denoted p1.2-Neo, p1.2-Zeo, and p1.2-Hygro, were used for insertion of the eGFP protein ORF. All three resulting plasmids showed similar transient transfection efficiencies in CHO DG44 cells (19–24% by electroporation), and the resulting cell populations were used to generate stably transfected cell populations in the suspension culture under variable selection pressures for each antibiotic used. The control plasmids p1.1eGFP and p1.1(EBVTR-)eGFP were transfected using the same procedure and stably transfected cell populations were generated by suspension cultivation in the presence of 200 nM MTX. The populations obtained were examined to determine the proportion of eGFP-expressing cells and eGFP levels in cell lysates (Figure 3). We found that for all three selection markers at all levels of drug selection pressure the resulting cell populations contained more than 75% of eGFP-positive cells. For the hygromycin and MTX resistance markers, less than 5% of the cells were eGFP-negative. The level of eGFP in the cell lysates was maximal for hygromycin selection, peaking as 8.9% of the total cellular protein with 0.5 mg/ml of hygromycin. In contrast, eGFP levels in the polyclonal cell populations obtained from transfection with p1.1eGFP or p1.1(EBVTR-)eGFP were much lower at 1.9% and 1.0%, respectively; however, eGFP expression levels for the p1.1 vector could potentially increase by eight-fold using the MTX-driven target gene amplification described above.We also measured the intracellular eGFP distribution in polyclonal cell populations using FACS (Figure 5). Virtually no cells were eGFP-negative with DHFR and hygromycin selection markers, whereas with the neomycin resistance gene the level of eGFP-negative cells was inversely proportional to the concentration of G418 used. The mean eGFP level for the upper 10% of the eGFP-positive cells was not dependent on the antibiotic concentration for neomycin and zeocin selection, whereas with hygromycin selection the mean eGFP level was higher at higher antibiotic concentrations.

Analysis of the copy numbers of the genome-integrated plasmids using quantitative PCR revealed that the p1.2-Hyg-eGFP plasmid generated the maximum number of inserts, correlating with the highest expression level of eGFP. While the p1.2-Zeo-eGFP plasmid exhibited higher eGFP expression levels than p1.2-Neo-eGFP, it was present at half the copy number. In the case of plasmids containing the DHFR selection marker, the presence of the EBVTR element resulted in higher eGFP expression levels at lower numbers of genome inserts; this probably indicates that EBVTR drives integration events in areas of the genome that are transcriptionally active.

## Conclusions

Creation of mammalian cell lines that express high levels of recombinant protein and maintain stable production levels over several months of cultivation is still a very time-consuming and labour-intensive process. Introduction of EEF1A-based vectors superseding CMV-based types has enabled smaller numbers of cell clones to be screened and evaluated by increasing the mean level of target protein expression. We have modified existing EEF1A-based vectors by linking the DHFR selection marker and target gene in the bicistronic RNA, shortening the overall plasmid size, and adding an EBVTR element. The presence of an EBVTR element in the resulting p1.1 vector increased the stable transfection rate by a factor of 24, and increased the target protein expression level by eight-fold using a single round of MTX-driven target gene amplification. Two consecutive rounds of MTX-driven amplification, performed for suspension culture, resulted in the polyclonal cell population with the eGFP expression level comprising 9.0% of the total cytoplasmic protein. Compatible vectors bearing antibiotic resistance markers instead of the DHFR gene were created and found to be approximately equal to the DHFR-based vector for generation of highly productive cell populations. We found that the EEF1A-based vector, p1.2-Hygro, containing the hygromycin selection marker, allowed direct generation of a polyclonal cell population that was nearly devoid of eGFP-negative cells, while eGFP expression comprised up to 8.9% of the total cytoplasmic protein. This level of eGFP expression corresponds to only 30 copies of the target gene per single haploid genome, in contrast to CMV-based vectors that have thousands of copies per genome in highly productive lines [19]. The set of vectors developed herein allows generation of highly productive and stable cell clones with limited effort and such vectors may be employed to create cell lines for production of biosimilar pharmaceuticals. p1.1 or p1.2-based plasmids, stably transfected into polyclonal cell populations expressing large quantities of target proteins at a scale of 4–6*10^7^ cells, can be generated in less than one month by simple periodic passage of a culture from a shaking flask. This technique may be useful for obtaining milligram quantities of mutants of a protein of interest or for evaluation of several mAb clones. Cells from these polyclonal populations may be also used for direct development of industrially applicable clonal cell lines by limiting dilution.

## Abbreviations

CHO cells: Chinese hamster ovary (CHO) cells; MTX: Methotrexate; eGFP: Enhanced green fluorescent protein; EBVTR: Concatemer of the terminal repeats from the Epstein-Barr virus; PCR: Polymerase chain reaction; Kbp: Kilo base pairs (1000 base pairs); EEF1A: Chinese hamster Elongation factor 1 alpha; IRES: Internal ribosome entry site; DHFR: dihydrofolate reductase (EC 1.5.1.3); ORF: open reading frame; RFU: relative fluorescence units.

## Competing interests

JAH, AGG and KGS declare that they have no competing interests. NAO, SVK and IIV are inventors of the patent RU2488633, which covers use of the p1.1 vector.

## Authors’ contributions

NAO developed the experimental strategy and plasmid design, performed cloning procedures, and drafted the manuscript. SVK performed the cell culture experiments and helped to draft the manuscript. IIV initiated the project, participated in its design and coordination and drafted the manuscript. NAO, JAH and IIV conducted the copy number determination experiments, SVK, JAH and IIV performed eGFP level determinations. AGG and KGS coordinated the project. All of the authors have read and approved the final manuscript.

## Supplementary Material

Additional file 1 Primer sequences used for cloning and sequencing the p 1.1 and p 1.2 expression vectors. Click here for file
