# Supplementary material for: Improved elongation factor-1 alpha-based vectors for stable high-level expression of heterologous proteins in Chinese hamster ovary cells
Source: BMC Biotechnol. 2014 Jun 14;14:56. doi: 10.1186/1472-6750-14-56 (PMC4067061; doi:10.1186/1472-6750-14-56)
Supplement: Additional file 1 — Primer sequences used for cloning and sequencing the p 1.1 and p 1.2 expression vectors. [file 1472-6750-14-56-S1.pdf]

SUPPLEMENTARY TABLES

Table S1. Primer sequences used for cloning the p 1.1 and p 1.2 expression vectors. F = forward primer; R = reverse primer.

Name	Primer sequence 5′ to 3′
**pBL-plasmids inverse PCR primers**	
IP-PL-F	AAGCTAGCGGCCGCGGATCCCGGGTCTAGACATATGTCGACCTCTAGCTAGAGCTTGGCGTAATCATGGTCATAGC
IP-PL-R	TCGCGATACGTACCTCGAGGTTATAAGAATTCTGCAGGTGGCACTTTTCGGG
IP-TFpBL-F	GGCGCGCCATGGCTCTTCCGCTTCCTC
IP-TFpBL-R	GAGCTCCGGACGTCGACATATGTCTAG
**sequencing primers forpBL-plasmids**	
SQ-BlaN-F	TAAGGGCGACACGGAAATG
SQprimer	CGCTTCCTCGCTCACTGACT
**EBV terminal repeat fragment construction**	
EBV-H1F1-F	AATTCATGCATGGCAATGGAGCGTGAAGAAGCGCCCCAGGGCTGACCCCGGCAAACGTGAC
EBV-H1F1-R	CCGGGTCACGTTTGCCGGGGTCAGCCCTGGGGCGCTTCTTCACGCTCCATTGCCATGCATG
EBV-H1F2-F	CCGGGGCTCCGGGGTGACCCAGGCAAGCGTGGCCAAGGGGCCCGTGGGTGACACAGGCAACCCTGACAAAGGCCCCCCAGGAAAGACCC
EBV-H1F2-R	CCGGGGGTCTTTCCTGGGGGGCCTTTGTCAGGGTTGCCTGTGTCACCCACGGGCCCCTTGGCCACGCTTGCCTGGGTCACCCCGGAGCC
EBV-H1F3-F	CCCGGGGGGCATCGGGGGGGTGTTGGCGGGTCATGGGGGGGGCGGGTCATG
EBV-H1F3-R	CGCGGCATGACCCGCCCCCCCCATGACCCGCCAACACCCCCCCGATGCCCCCCGGGT
EBV-H1F4-F	CCGCGCATTCCTGGAAAAAGTGGAGGGGGCGTGGCCTTCCCCCCGCGGCCCCCTAGGA
EBV-H1F4-R	CCTAGGGGGCCGCGGGGGGAAGGCCACGCCCCCTCCACTTTTTCCAGGAATG
EBV-H2F1-F	GCTAGCCCCCCCGCAGAGAGCGGCGCAACGGCGGGCGAGCGGCGGGGGGTCGGGGTCCGCGGGCTCCGGAA
EBV-H2F1-R	ACCGGAGCCCGCGGACCCCGACCCCCCGCCGCTCGCCCGCCGTTGCGCCGCTCTCTGCGGGGGGGCTAGCA
EBV-H2F2-F	CCGGGGGCTGCGGGCGGTGGATGGCGGCTGGCGTT
EBV-H2F2-R	CCGGAACGCCAGCCGCCATCCACCGCCCGCAGCCC
EBV-H2F3-F	CCGGGGATCGGGGGGGGGTCGGGGGGCGCTGCGCGGGCGCAGCCATGCGTGACCGTGATGAGATGCATG
EBV-H2F3-R	AATTCATGCATCTCATCACGGTCACGCATGGCTGCGCCCGCGCAGCGCCCCCCGACCCCCCCCCGATCC
EBV-H1F2-SHORTF	GGTGACCCAGGCAAGCG
EBV-H2F2-SHORTR	CAGCCGCCATCCACCG
EBV-H2F1-SHORTF	AGAGCGGCGCAACGGC

Name	Primer sequence 5′ to 3′
**DHFR ORF amplification**	
AD-DHFR-F	AAGATCTGCCACCATGGTTC
AD-DHFR-R	ATCTAGATTAGTCTTTCTTCTCGTAGAC
**Elongation factor 1A flanking regions modular assembly**	
AD-5CHEF1-R	ACCTAGGGTTCTCAGCCAGTCG
AD-5CHEF2-F	ATCTAGAAAATCTCTGTGAAAAAGG
AD-5CHEF2-R	AGGATCCAACTCAGAGTCAGG
AD-5CHEF3-F	TGGATCCTTTAGAGCTACATGG
AD-5CHEF3-R	TCCTAGGTAGACCAGGCTGACCTC
AD-5CHEF4-F	AACTAGTGAGTTTCAGGGCACC
AD-5CHEF4Xho-R	ACTCGAGATCCGGGTGTCCCTTC
AD-5CHEF1-F	AGAATCCCACGTTGTGCATAGAAACAG
AD-5CHEF5-F	AGTCGACACAGCCTTGGCAGTG
AD-5CHEF5-R	TCCATGGCCAGGCTGGCCTG
AD-5CHEF61-F	ATCATGAAAGTAATTCTTGGAATTTGCCCATTTTGAGTTTGGAGCGAAGCTGATTGACAAAGCTGC
AD-5CHEF61-R	CTTTGTCAATCAGCTTCGCTCCAAACTCAAAATGGGCAAATTCCAAGAATTACTTTCATGAT
AD-5CHEF62-F	TTAGCCGTTCAAAGGTATTCTTCGAACTTTTTTTTTAAGGTGTTGTGAAAACCTCGAGGA
AD-5CHEF62-R	TCCTCGAGGTTTTCACAACACCTTAAAAAAAAAGTTCGAAGAATACCTTTGAACGGCTAAGCAG
AD-3CHEF1-NheIF	AGCTAGCATATTACCCCTAACACCTGC
AD-3CHEF1-R	ATCTAGAGCAAAGCCTGCCTCA
AD-3CHEF2-F	AGCTAGCAATTTTAGAACTAGT
AD-3CHEF2-R	ACCTAGGCGTGACAAATTTTTC
AD-3CHEF3-F	AGCTAGCATTCAACTTAGTGGT
AD-3CHEF3-R	ACCTAGGGGGTTTGATTTCCC
AD-3CHEF4-F	ACCTAGGCCTTCTGTCAGTAA
AD-3CHEF4-R	AGAGCTCAAGAATAGCCTGGT
AD-3CHEF5-F	AGAGCTCAGATTAGCCTGTCT
AD-3CHEF5-R	TGGATCCTCCTGCCTCCAGAGACAAAAAC
AD-3CHEF6-F	ATGATCATTTGAGCATAGGGAG
AD-3CHEF6-XbaR	ACTCGAGTCACTTGCTGTTCTTCCAGA

Name	Primer sequence 5′ to 3′
**Sequencing primers for Elongation factor 1A flanking regions**	
SQ-5CH6-F	GCCGCTGCTTCCTGTGAC
SQ-3CH1-R	ACAAACAGTTCTGAGACCG
**Antibiotic cassettes amplification**	
AD-HYG-AscF	TGGCGCGCCTGTGGAATGTGTGTCAGTTAG
AD-HYG-AscR	TGGCGCGCCTAGAGGTCGACGGTATACAG
**Kozak-EGFP ORF amplification**	
AD-EG-AbsF	TTCCTCGAGGCCGCCACCATGGTGAGCAAGGGC
AD-EG-NheR	ATGCTAGCTTACTTGTACAGCTCGTC

Table S2. Primer sequences used for quantitative PCR. F = forward primer; R = reverse primer.

Name	Primer sequence 5′ to 3′
RT-GFP-F	GCACCATCTTCTTCAAGG
RT-GFP-R	TTGTGGCTGTTGTAGTTG
RT-HYG-F	TTCGGCTCCAACAATGTC
RT-HYG-R	GTCTGCTGCTCCATACAAG
RT-Neo-F	CCGTGATATTGCTGAAGAG
RT-Neo-R	GTCAAGAAGGCGATAGAAG
RT-Zeo-F	AGTTGACCAGTGCCGTTCC
RT-Zeo-R	GGCGAAGTCGTCCTCCAC
